# The Geospatial Distribution of Myositis and Its Phenotypes in the United States and Associations With Roadways: Findings From a National Myositis Patient Registry

**DOI:** 10.3389/fmed.2022.842586

**Published:** 2022-03-16

**Authors:** Md M. Hossain, Jesse Wilkerson, John A. McGrath, Payam N. Farhadi, Cole Brokamp, Md T. F. Khan, Bob Goldberg, Hermine I. Brunner, Maurizio Macaluso, Frederick W. Miller, Lisa G. Rider

**Affiliations:** ^1^Division of Biostatistics and Epidemiology, Cincinnati Children's Hospital and Medical Center, Cincinnati, OH, United States; ^2^Department of Pediatrics, University of Cincinnati College of Medicine, Cincinnati, OH, United States; ^3^Social and Scientific Systems, A DLH Holdings Corp Company, Durham, NC, United States; ^4^Environmental Autoimmunity Group, Clinical Research Branch, National Institute of Environmental Health Sciences, National Institutes of Health, Department of Health and Human Services, Bethesda, MD, United States; ^5^Kelly Government Solutions, Rockville, MD, United States; ^6^Division of Biostatistics and Bioinformatics, University of Cincinnati, Cincinnati, OH, United States; ^7^The Myositis Association, Alexandria, VA, United States; ^8^Division of Rheumatology, Cincinnati Children's Hospital Medical Center, Cincinnati, OH, United States

**Keywords:** myositis, prevalence, cluster, major or minor road networks, log-Gaussian Cox process

## Abstract

**Background:**

Little is known about the spatial distribution of idiopathic inflammatory myopathies (IIM) in the United States (U.S.), or their geospatial associations.

**Methods:**

We studied a national myositis patient registry, with cases diagnosed in the contiguous U.S. from 1985–2011 and comprised of dermatomyositis (DM, *n* = 484), polymyositis (PM, *n* = 358), and inclusion body myositis (IBM, *n* = 318) patients. To assess the association of myositis prevalence with distance from roads, we employed log-Gaussian Cox process models, offset with population density.

**Results:**

The U.S. IIM case distribution demonstrated a higher concentration in the Northest. DM, IBM, and cases with lung disease were more common in the East, whereas PM cases were more common in the Southeast. One area in the West and one area in the South had a significant excess in cases of DM relative to PM and of cases with lung disease relative to those without lung disease, respectively. IIM cases tended to cluster, with between-points interactions more intense in the Northeast and less in the South. There was a trend of a higher prevalence of IIM and its major phenotypes among people living within 50 m of a roadway relative to living beyond 200 m. Demographic characteristics, rural-urban commuting area, and female percentage were significantly associated with the prevalence of IIM and with major phenotypes.

**Conclusions:**

Using a large U.S. database to evaluate the spatial distribution of IIM and its phenotypes, this study suggests clustering in some regions of the U.S. and a possible association of proximity to roadways.

## Introduction

Idiopathic inflammatory myopathies (IIM) are rare, life-threatening, systemic autoimmune disorders characterized by chronic proximal muscle inflammation and weakness. The most common IIM are dermatomyositis (DM) with characteristic skin rashes of Gottron's papules and heliotrope rash, polymyositis (PM) with an immune attack on myofibers, and inclusion body myositis (IBM), with progressive weakness and muscle atrophy in older patients (1). The overall prevalence of IIM has been estimated to range from 14.0–21.4 cases per 100,000 population in the United States (U.S.) (2–5). Among the subgroups, the prevalence of DM has been reported as 13 cases (6) and for IBM as 5.05 cases (7) per 100,000 population. Prevalence estimates have varied, depending on the database utilized, enrollment in health systems, and coding of IIM diagnoses.

Little is known about the spatial distribution of IIM and its associations with environmental exposures (8). There is strong empirical evidence that environmental causes contribute to the development of systemic autoimmune diseases (9–11). For rheumatoid arthritis, significant regional differences have been identified in the geospatial distribution (12–14). European data suggest a latitudinal gradient of IIM, with increased risk of DM relative to PM in southern Europe, likely related to increased exposure to ultraviolet B radiation (15). An association of ultraviolet radiation exposure with DM has also been suggested in other studies (16, 17).

Adult rheumatoid arthritis and juvenile idiopathic arthritis prevalence have both been linked to exposure to air pollution, identified by residential location near airports or road networks (18–21). Only one study has examined air pollution as a risk factor in myositis, and found a residential association with air emissions in clinically-amyopathic DM, but not classic DM (22). Exposure to air pollutants and tobacco smoking during fetal development may contribute to juvenile DM risk (23). Increasing risk of PM was also suggested for tobacco smoking in Caucasians (24).

To further evaluate the relevance of environmental exposures to the development of IIM, we aimed to examine the geospatial characteristics of IIM and its subgroups in the U.S. using a national patient registry. The geocoded locations of cases were analyzed using the methods developed in spatial point processes. We also examined whether the prevalence rates of IIM and its subgroups are associated with distance to roadways, with the hypothesis that air pollution exposure from road traffic may be involved in pathogenesis.

## Materials and Methods

### Study Population, Design, and Data Source

We extracted data from a U.S. national myositis patient registry database, called ‘MYOVISION' (17, 25) containing IIM patients who enrolled between December 2010 and July 2012 by completion of a patient questionnaire after signing informed consent. The present study cohort consisted of 1,247 adult and juvenile DM, PM, and IBM cases, diagnosed between 1985 and 2011, residing in one of the 48 contiguous U.S. states where road network locations were available. The present study was restricted to patients who had the same residential zip code at diagnosis and enrollment (Table 1).

**Table 1:** Demographic and clinical characteristics of the myositis study cohort (*n* = 1,247).

**Demographic and clinical characteristics**		**Minimum distance from the residential zip code to the major or minor road networks (in m)**		
	**All (*n* = 1,247)**	** <50 m (*n* = 61)**	**50–199 m (*n* = 136)**	**≥200 m (*n* = 1,050)**
	***n* (%)**	***n* (%)**	***n* (%)**	***n* (%)**
Diagnosis age (years)^*^				
• 0–6	42 (3)	0 (0)	4 (3)	38 (4)
• 7–17	45 (4)	5 (8)	6 (4)	34 (3)
• 18–44	339 (27)	12 (20)	38 (28)	289 (28)
• 45–64	631 (51)	29 (48)	58 (43)	544 (52)
• ≥65	190 (15)	15 (25)	30 (22)	145 (13)
Sex				
• Female	877 (70)	41 (67)	97 (71)	739 (70)
• Male	370 (30)	20 (33)	39 (29)	311 (30)
Ever smoke				
• No	788 (63)	43 (70)	80 (59)	665 (64)
• Yes	454 (37)	18 (30)	56 (41)	380 (36)
Race/ethnicity				
• Non-Hispanic White	1,106 (89)	56 (92)	119 (88)	931 (89)
• Other Race	141 (11)	5 (8)	17 (13)	119 (11)
Education level				
• < High school	14 (1)	1 (2)	1 (0.7)	12 (1)
• High school graduate	288 (23)	21 (34)	23 (17)	244 (23)
• College graduate	483 (39)	17 (28)	55 (40)	411 (39)
• Unknown	462 (37)	22 (36)	57 (42)	383 (36)
Median household income^*^				
• < $60,000	584 (47)	40 (66)	69 (51)	475 (45)
• ≥$60,000	653 (52)	20 (33)	67 (49)	566 (54)
• Unknown	10 (0.8)	1 (2)	0 (0)	9 (0.9)
Rural-urban commuting area (RUCA)^*^				
• Metropolitan (RUCA ≤ 3)	1,022 (82)	39 (64)	106 (78)	877 (84)
• Non-metropolitan (RUCA ≥ 4)	203 (16)	20 (33)	29 (21)	154 (15)
• Unknown	22 (2)	2 (3)	1 (0.7)	19 (2)
Myositis Subtype				
• Dermatomyositis (DM)	484 (39)	17 (28)	56 (41)	411 (39)
• Juvenile DM^$^	82 (7)	4 (7)	10 (7)	68 (6)
• Inclusion Body Myositis (IBM)	318 (26)	21 (34)	34 (25)	263 (25)
• Polymyositis (PM)	358 (29)	18 (29)	36 (27)	304 (29)
• Juvenile PM^$^	5 (0.4)	1 (2)	0 (0)	4 (0.4)
Lung disease				
• Yes^#^	303 (24)	16 (26)	39 (29)	248 (24)
• No^#^	918 (74)	45 (74)	95 (70)	778 (74)
• Unknown	26 (2)	0 (0)	2 (1)	24 (2)

* *Statistical significance at p <0.05*.

$ *Juvenile DM and Juvenile PM cases excluded in all subgroup analyses*.

# *IIM cases with and without lung disease include juvenile IIM cases*.

### Statistical Analysis

#### Geospatial Distribution

**Figure 1:**
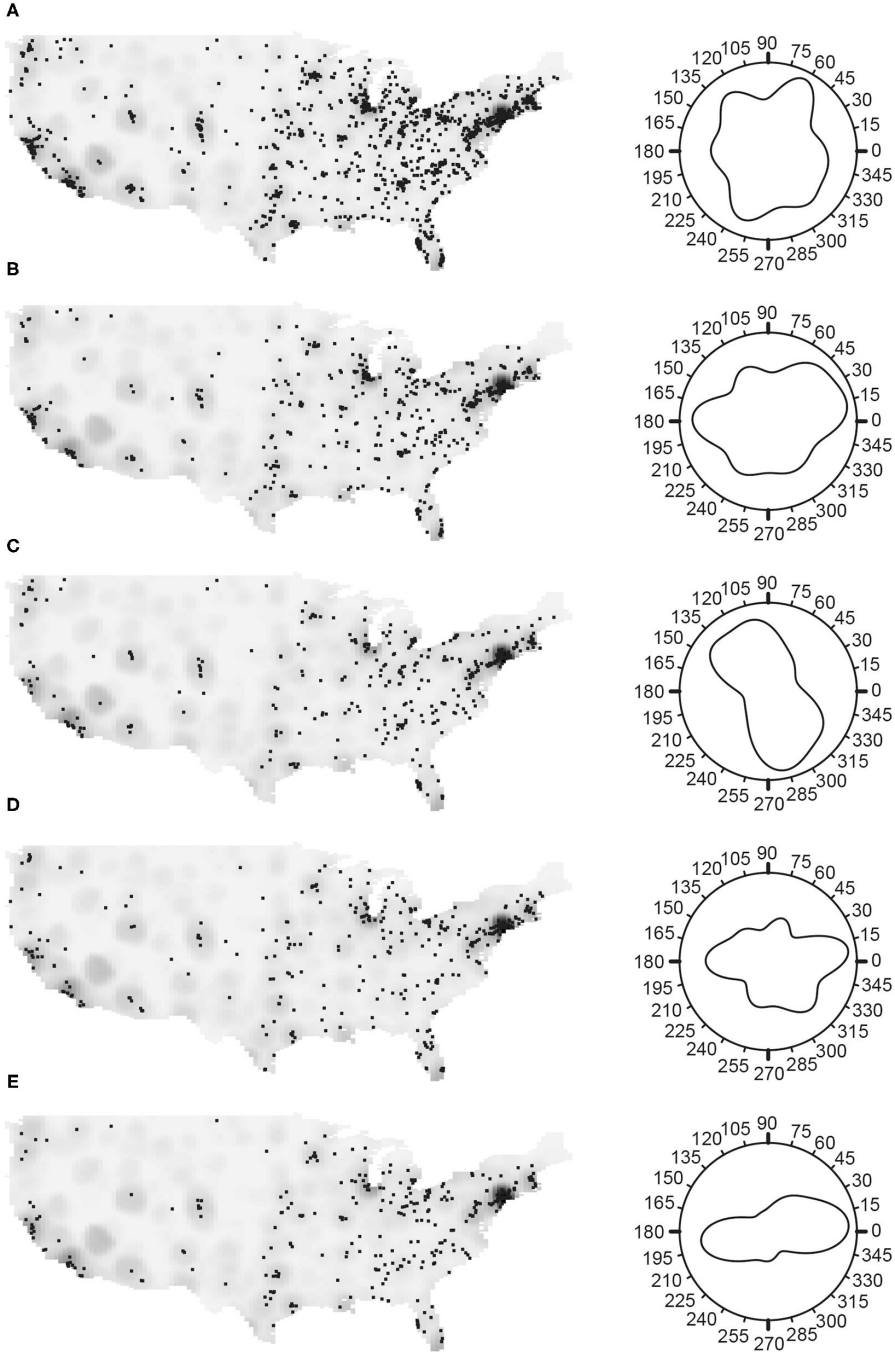
The spatial distribution of myositis (IIM) cases in the MYOVSION National Myositis Patient Registry. The left panels show cases as black dots with population density in the gray background for the average U.S. population based on 2000 and 2010 censuses by census tract. In the right panels, Rose diagrams for the angular density of myositis cases using the counterclockwise from east convention. **(A)** All IIM; **(B)** Dermatomyositis; **(C)** Polymyositis; **(D)** Inclusion body myositis; and **(E)** IIM cases with lung disease.

We used ArcGIS (26) to geocode the latitude-longitude coordinates of the zip code centroid of each patient's residence at diagnosis, to map the prevalence of the study population across the U.S. (Figure 1A). We treated case location (longitude and latitude) as a spatial point process (see Myositis Supplement Methods for details), employing the inhomogeneous *J*-function (27, 28) to examine whether there is any clustering of myositis cases. Directions of higher concentration of IIM cases and its subgroups (DM, PM, IBM) were determined using the nearest-neighbor orientation density methods (29). Spatially adaptive bandwidth selection methods were used to address inhomogeneity in the distribution of myositis cases when evaluating spatial trends in increased prevalence (30) relative to other subgroups. The independence of IIM subgroups was tested using the mark connection function (31). Space-time separability tests (31, 32) were used to evaluate the independence of spatial and temporal processes in generating cases. Based on the tests of separability between the spatial point and temporal processes using the nearest-neighbor and variogram, we concluded that a spatial point process was appropriate for the entire study period.

#### Air Pollution Exposure

Since the location of IIM cases and subgroups suggested various patterns of clustering, the log-Gaussian Cox process (LGCP) (33) with population density as an offset was used for assessing the effect of the air pollution exposure surrogate, distance to roads, on the prevalence of IIM cases. Two separate LGCP models, unadjusted and adjusted, were fitted first for all IIM cases, then separately for DM, PM, IBM, and for IIM cases with and without lung disease, since the spatial distributions of cases and subgroups were independent. Unadjusted models included only distance from roads as the independent variable, while adjusted models added all covariates as detailed below. The fitted LGCP models were examined using two standard approaches: (1) assessing goodness-of-fit by Monte Carlo tests; and (2) examining whether the empirical *J*-function lies within the 95% confidence band for the model estimated *J*-function (27, 28). Exposure to traffic-related air pollution was approximated by the minimum distance from the residential zip code at diagnosis to major or minor road networks. We used the Topologically Integrated Geographic Encoding and Referencing (TIGER) shape files from the 2010 U.S. census to define major and minor road networks (https://catalog.data.gov/dataset/tiger-line-shapefile-2010-series-information-file-for-the-2010-census-block-state-based-shapefi). The minimum distance to a road network was categorized as <50 m, 50–199 m, and ≥200 m. In assessing the effect of living in the <50 m or 50–199 m distance categories, we used the distance ≥200 m as a reference category.

#### Covariates

Geographic and demographic variables were considered as covariates. The geographic variables are the longitude and latitude of the residential zip code centroid where the patient was living at diagnosis. Demographic variables included census tract level measures of rural-urban commuting area (RUCA) codes, median household income, female percentage of population, percentage of population by age groups (7–17 years, 18–44, 45–64, and 65 years or older), and white percentage of population; and county level estimates of smoking percentage of population. The RUCA code was acquired at the census-tract level for the census years 2000 and 2010 from the U.S. Department of Agriculture (https://www.ers.usda.gov/data-products/rural-urban-commuting-area-codes.aspx). The county level estimates of cigarette smoking prevalence for the years 2000 and 2010 were acquired from a study (34) that used the data from Behavioral Risk Factor Surveillance System. All other demographic variables were acquired from the U.S. Census Bureau for the same census years. We averaged the demographics over the census years to get a unique value for each census tract (or, county) representing the whole study period represented by the patients' diagnosis dates.

For all computation and graphics, we used the R computing software (R Foundation, Vienna, Austria) (35): the “spatstat” R package (36) computes statistics related to spatial point processes such as nearest-neighbor orientation density, mark connection function, *J*-function, and fits LGCP models, and the “sparr” R package (37) computes areas of increased prevalence of cases relative to other subgroups.

## Results

Registry participants (*n* = 1,247) were primarily female (70%), non-smoker (63%), non-Hispanic white (89%), living in metropolitan areas (82%), without lung disease (74%), in the 45–64 year old age group (51%), and had a median household income of >$60,000 (52%) (Table 1). The distribution of the study cohort by sex (*p* = 0.350), ever smoking status (*p* = 0.276), race/ethnicity (*p* = 0.782), and myositis subtypes (*p* = 0.455) did not appear to differ by distance to road networks (Table 1). A greater percentage of patients with median household income < $60,000 were living close to major or minor road networks, whereas those with >$60,000 were living further away (*p* = 0.012) (Table 1). Similar trends were also observed for metropolitan and non-metropolitan areas (*p* = 0.0015).

In general, the density of IIM cases and its subgroups positively correlated with the general population density (Figure 1). To examine whether a higher myositis prevalence existed in any direction, the Rose diagram in Figure 1A (Supplementary Table S1) shows the nearest-neighbor orientation density curve, using the counterclockwise from east convention. There were more IIM cases located in the East, particularly in the Northeast, and fewer cases in the Midwest region (Figure 1A), this peak maximized at 63.4°. Among the subgroups, DM and IBM were more common in the East (Figures 1B,D, with maximums at 24.0° and 357.2°, respectively) and PM was more common in the Southeast, with the maximum at 292.4° (Figure 1C). Patients with and without lung disease have higher prevalence in the East (Figure 1E, maximum at 8.5°, and Supplementary Table S1, maximum at 336.8°, respectively) similar to those of DM and IBM patients.

The number of myositis cases per year varied from 5 (in years 1986 and 1987) to 95 (in year 2001), with a mean of ~46 cases per year (SD: 32.5) and a median of 50 (IQR: 15.5–75) (Supplementary Figure S1).

**Figure 2:**
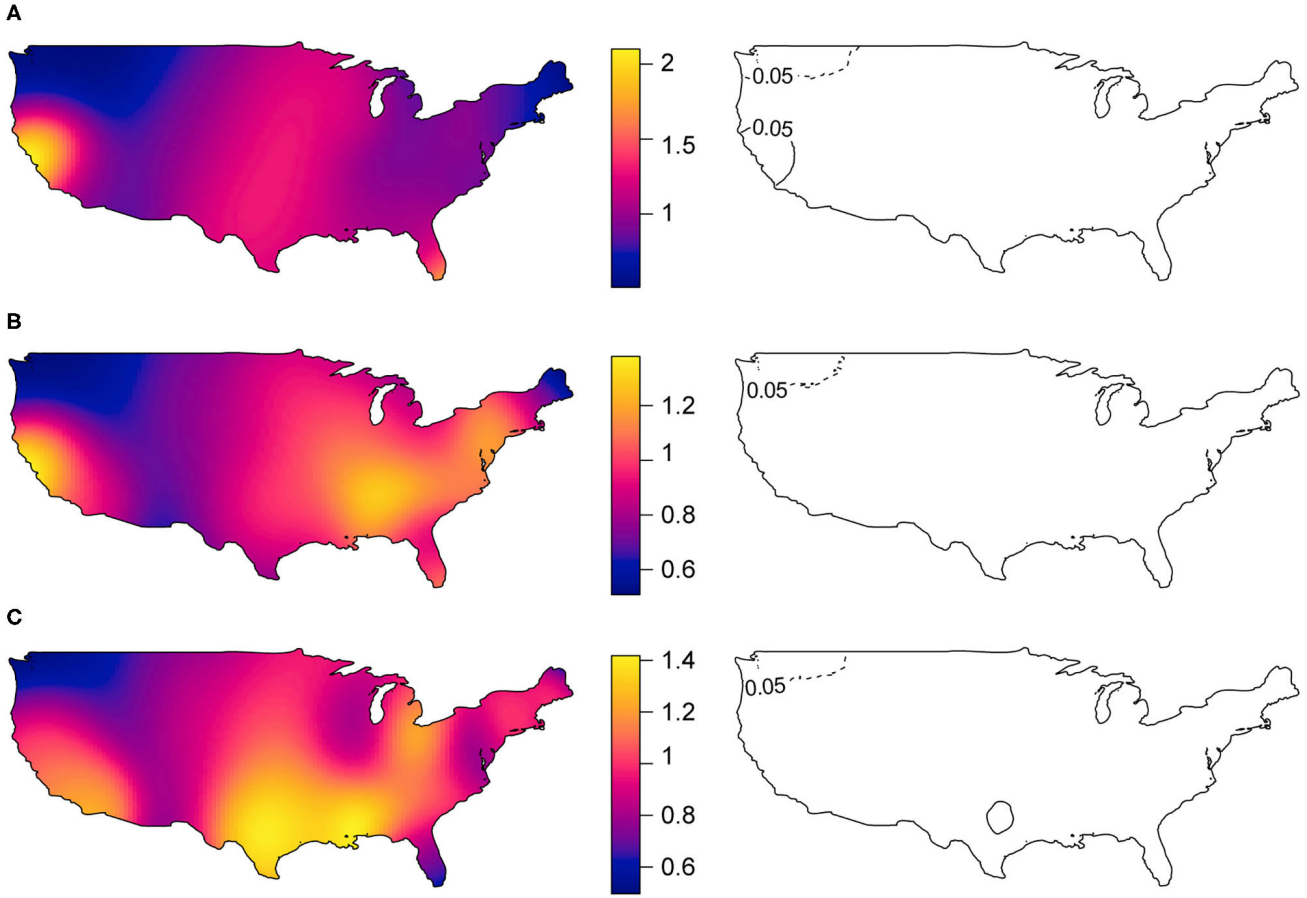
Spatial distribution of myositis subgroup relative prevalence of cases (in left panels) and statistically significant areas with excess and lower prevalence of cases (in right panels). **(A)** Dermatomyositis vs. Polymyositis cases, **(B)** Dermatomyositis vs. Inclusion body myositis cases, and **(C)** IIM cases with lung disease vs. IIM cases without lung disease. Areas with black solid lines and with dotted lines showing statistically significant increased and decreased prevalence of cases at 5% level-of-significance, respectively.

The spatial distribution of increased prevalence of DM cases relative to PM cases (Figure 2A) shows an area in the West (San Francisco Bay area) and an area in the Northwest of the U.S. (Seattle-Tacoma-Bellevue Metro Area) that had statistically significant excess and lower prevalence of cases, respectively. The Seattle-Tacoma-Bellevue Metro Area also had statistically significant lower prevalence of cases of DM relative to IBM (Figure 2B). Similarly, relative to cases without lung disease (Figure 2C), the Dallas-Fort Worth-Arlington Metro Area within Texas and the Seattle-Tacoma-Bellevue Metro Area within Washington had a statistically significant excess and lower prevalence of cases with lung disease, respectively. There were no areas with significant excess or lower prevalence of PM cases relative to IBM.

**Figure 3:**
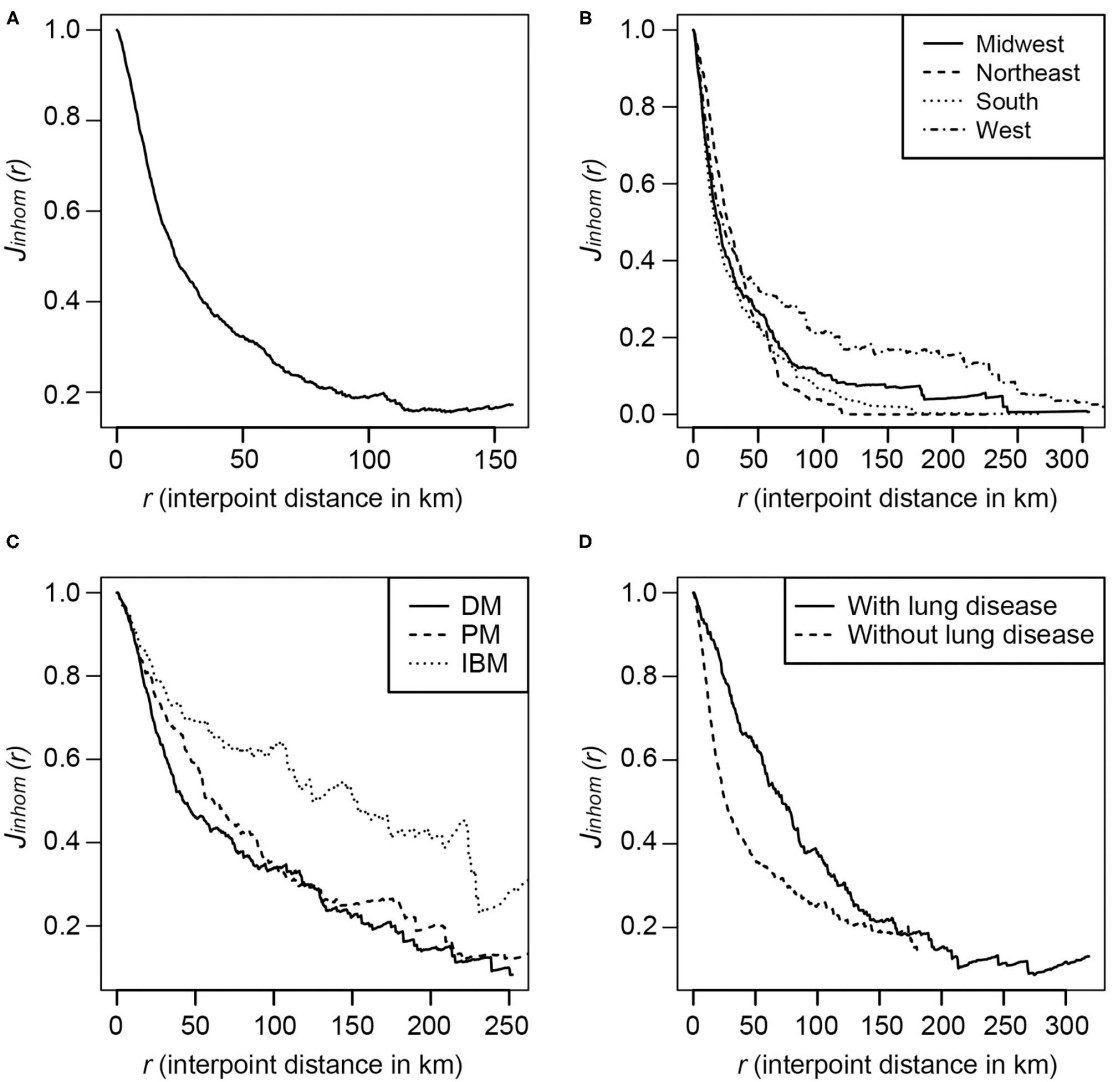
The inhomogeneous J-function estimates for the incidence of myositis in the U.S.in the MYOVISION National Myositis Patient Registry. **(A)** All IIM cases; **(B)** By region for 48 contiguous U.S. states: Midwest, Northeast, South and West, according to the definition from U.S. Census Bureau; **(C)** By IIM subgroups: Dermatomyositis, Polymyositis, and Inclusion body myositis; **(D)** IIM cases with and without lung disease.

The inhomogeneous J-function estimates for the myositis cases in the U.S. after adjusting for population density (Figure 3A) were consistently <1, indicating that the myositis cases in the U.S. have a clustering pattern. The curve was almost flattened after around 120 km, indicating that the interaction between points is limited at greater distances. This clustering pattern varied by geographic region (Figure 3B), with the curves for the Northeast flattening around 65 km and for the West around 240 km. Similarly, the clustering patterns varied among myositis subgroups (Figure 3C), with interpoint distances of DM and PM cases differing from IBM cases, and with interpoint distances differing between cases with lung disease compared to cases without lung disease (Figure 3D).

The average density of IIM was ~3.8 cases per 10,000 square miles within the 48 contiguous U.S. states, and by subgroups, the density of cases per 10,000 square miles was 1.6 for DM, 1.2 for PM, and 1.0 for IBM. There were 1.0 cases with lung disease per 10,000 square miles and 2.9 cases without lung disease. After adjusting for the effect of average population density in an LGCP model, the density of IIM was ~2.6 per 10,000 square miles and the prevalence of IIM was ~2.8 cases per 1 million population. These estimates from LGCP model have taken into consideration that the areas with high population densities are also the areas of high IIM cases. The prevalence of myositis subgroup cases per 1 million population within the 48 contiguous U.S. states was 1.1 for DM, 0.8 for PM, 0.7 for IBM, and 0.7 for IIM with lung disease and 2.0 for IIM without lung disease.

Subgroup analyses included patients with DM, PM, IBM, and patients with and without lung disease (Supplementary Figure S2). Since the four estimated mark connection functions for the pairs DM and PM (A), DM and IBM (B), PM and IBM (C), and IIM with and without lung disease (D) are within the bounds (shaded area) created by Monte Carlo simulation, this indicates that the spatial distributions of case subgroups were independent of each other.

The relative changes in log-intensity of IIM cases due to minimum distance from the residential location at the time of diagnosis to the major or minor road network, after adjusting for population density as an offset value, are shown in Table 2. From the unadjusted model for all IIM, the prevalence of cases was 2.9 times higher if living within 50 m of major or minor road networks compared to living outside of 200 m. After adjustment for geographic and demographic covariates, the prevalence of IIM cases was 3.3 times higher among those living within 50 m of the road network. The adjusted effect of living within 50 m of a road network from the subgroup analysis ranged from a 2.2-fold increase in prevalence for DM to a 6.5-fold increase in prevalence for IBM. For both unadjusted and covariate adjusted models, the effect estimates for the minimum distance of living from the road network compared to living ≥200 m were statistically insignificant. The coefficient estimates with 95% CI for all covariates in adjusted models for all IIM and subgroups are presented in Supplementary Table S2, indicating that RUCA and percentage of females have significant negative and positive effects, respectively, on the prevalence of IIM cases. While for major phenotypes, the percentage of females had significant negative effects on the prevalence of DM cases and cases with and without lung disease, whereas RUCA had significant positive effects on the prevalence of IBM cases and cases with lung disease. The model goodness-of-fit test for all myositis incidences from the global Monte Carlo test was significant (*p* = 0.002), indicating an overall good fit of the LGCP model to all IIM cases. The model validation results for myositis subtypes are very similar (Supplementary Figure S3).

**Table 2:** The unadjusted and adjusted relative risk estimates (with 95% CI) of the minimum distance from the residential location at diagnosis to major or minor road networks of developing IIM (from the log-Gaussian Cox process (LGCP) model).

**Type of myositis**	**Minimum distance to road network**	**Unadjusted model^∧^**		Adjusted model^∧^^*^	
		**Relative risk estimate**	**(95% CI)**	**Relative risk estimate**	**(95% CI)**
All IIM	<50 m	2.93	(0.36, 23.49)	3.28	(0.27, 39.80)
	50–199 m	1.42	(0.43, 4.68)	1.17	(0.25, 5.50)
	≥200 m	1.00 (REF)		1.00 (REF)	
DM	<50 m	1.95	(0.09, 44.04)	2.19	(0.05, 87.54)
	50–199 m	1.38	(0.33, 5.79)	0.96	(0.13, 7.04)
	≥200 m	1.00 (REF)		1.00 (REF)	
PM	<50 m	2.94	(0.17, 51.33)	3.39	(0.12, 93.71)
	50–199 m	0.98	(0.20, 4.69)	0.84	(0.10, 6.87)
	≥200 m	1.00 (REF)		1.00 (REF)	
IBM	<50 m	5.93	(0.67, 52.79)	6.54	(0.53, 80.87)
	50–199 m	1.52	(0.43, 5.36)	1.16	(0.23, 5.71)
	≥200 m	1.00 (REF)		1.00 (REF)	
Lung disease	<50 m	2.44	(0.13, 46.90)	2.69	(0.09, 81.01)
	50–199 m	2.24	(0.68, 7.39)	1.60	(0.36, 7.16)
	≥200 m	1.00 (REF)		1.00 (REF)	
No lung disease	<50 m	3.63	(0.39, 33.73)	4.06	(0.28, 59.60)
	50–199 m	1.18	(0.31, 4.47)	0.92	(0.14, 6.24)
	≥200 m	1.00 (REF)		1.00 (REF)	

∧ *In both LGCP models, population density was an offset value*.

* *Adjusted for longitude, latitude, rural-urban commuting area (RUCA), median household (MH) income, percent female, age groups, percent white, and percent smoking*.

*IIM, idiopathic inflammatory myopathies; DM, adult dermatomyositis; PM, adult polymyositis; IBM, inclusion body myositis; m, meters; CI, confidence interval*.

## Discussion

Based on the residential data of a large U.S. national myositis patient registry, the spatial distribution of myositis cases and the population density in the U.S. have a similar pattern. After adjusting for population density, however, we consistently observed that the myositis cases have clustering patterns for all IIM, by regions, and by subgroups. The prevalence of myositis cases is higher in the Northeast of the U.S. Interestingly, the Northeast region has also seen a higher incidence of Lyme disease, and a previous study suggested that myositis can be caused by Lyme Borreliosis (38). As blood samples to confirm this etiology through serologic testing were not available in this patient questionnaire registry, subsequent studies could examine Lyme as a potential etiologic factor. The IIM subgroups DM, IBM, as well as patients with lung disease were more common in the East, whereas PM was more common in the Southeast. Moreover, when we made relative comparisons of case prevalence among the subgroups, an area in the West, located in the San Francisco Bay Area had significant excess cases of DM relative to PM. Earlier studies suggested that the increased prevalence of case of DM relative to other types of IIM was possibly related to increased exposure to ultraviolet radiation (15, 17), but the reasons for the clustering seen in this study are not clear and require further investigation. In addition, we also observed an area in the South, located in the Dallas-Fort Worth-Arlington Metro Area with a statistically significant excess prevalence of cases with lung disease relative to cases without lung disease. According to the American Lung Association, the Dallas-Fort Worth-Arlington Metro Area ranked 17^th^ for high ozone days out of 229 metropolitan areas, 42^nd^ for 24-h particle pollution out of 216 metropolitan areas, and 50^th^ for annual particle pollution out of 204 metropolitan areas (39). We suspect that these exposures, but possibly multiple other environmental factors, may relate to the excess prevalence of cases in certain regions.

The higher prevalence of myositis cases in the Northeast is probably suggesting that the interactions between points are more intense in that region than in the South. Urbanization may be a contributing factor for the increased number of cases in certain areas. From all adjusted LGCP models, we observed a statistically significant negative effect for the RUCA, implying that rural areas had a lower prevalence of cases. As might be expected from the female predominance in myositis, we also observed a positive effect for female presence, meaning areas with a high percentage of females in the population have a higher prevalence of myositis cases in the MYOVISION study sample. These results are also reflected in Table 1, in that cases from metropolitan areas and with high proportions of females are predominant.

This study has several limitations, including first, the use of a convenience sample of U.S. IIM cases that may be non-representative of the general myositis population in the U.S. This may be reflected in our prevalence estimate, which was underestimated compared to an earlier published meta-analysis result with global data that the prevalence of inflammatory myopathies ranges from 2.4 to 33.8 per 100,000 population (40). The incidence data by year may not reflect actual changes in the annual incidence of myositis, but rather a bias toward enrollment of more recently diagnosed patients and inadequate representation in the number of cases. Another limitation is the lack of specific residential street addresses (due to privacy concerns) that would have allowed us to specify latitude and longitude coordinates. However, by socioeconomic and demographic characteristic such as urbanization and gender, this study population was similar to two earlier studies (2, 3).

A second limitation is the use of patient registry data, with a study questionnaire completed by enrolled patients that did not include physician or medical record confirmation. In addition, serum samples were not available for myositis autoantibody testing. The study design, therefore, limited our ability to discern certain subgroups of myositis. The PM subgroup, for example, may include patients with anti-synthetase syndrome and immune-mediated necrotizing myopathies (1), and thus, the spatial distribution of PM, as reported in this study, may not be fully accurate. In addition, from the patient questionnaire data, it cannot be determined if patient-reported lung disease is specifically interstitial lung disease, which would have greater prognostic importance (1). These issues should be further addressed in a subsequent study.

Another limitation may be that only 2010 road network data was used for calculating the distance from the residence zip code. The major and minor road network system in the 48 contiguous states of the U.S. probably did change during the patients' years of diagnosis (1985–2011), but it was difficult to define an average road map for this large time period. Presumably, road networks for the census years 1990 and 2000 were less dense than noted in the census year 2010, and therefore we may have overestimated exposure levels at the time of diagnosis. The census tract level covariates were derived from the averages of 2000 and 2010 census data. The estimates of exposure effect were sensitive to cut-points used for defining exposure categories. However, the cut-points were determined with the consultation of subject matter experts and from the literature (21). Our data also showed the distribution of cases (in percentages) over exposure categories by demographic and myositis subgroups to be almost equal. Also, the study may have been underpowered to detect differences. For example, a relatively low percentage of patients lived within 50 m of a roadway network. We also did not examine other sources of pollution, such as industrial emissions, but plan to examine this in a subsequent study. Finally, although air pollution correlates with proximity to roadways, additional influential exposures, such as noise levels, lack of greenery, stress and income levels may also correlate with roadway density (41).

This is the first national level study using a large U.S. database and spatial point process modeling techniques to illustrate the spatial characteristics that could be related to potential risk factors for myositis and its subgroups. Although application of spatial point processes is more common in ecology (42), it is an emerging tool in epidemiological research (43). We observed clustering of IIM and its phenotypes in some regions, and a statistically nonsignificant effect of distance from road network on myositis prevalence, but a significant effect of RUCA, possibly indicating exposure to higher level of air pollutants, consistent with previous studies that also found a significant correlation between clinically amyopathic DM with airborne pollutants (22) and urban dwellings (7). More research is needed to understand the role of proximity to roadways and air pollutants, as well as other exposures and risk factors, in the development of myositis, given the geospatial distribution of myositis patients nationwide.

## Data Availability Statement

The raw data supporting the conclusions of this article will be made available by the authors, without undue reservation.

## Ethics Statement

The studies involving human participants were reviewed and approved by Cincinnati Children's Hospital and Medical Center. The patients/participants provided their written informed consent to participate in this study.

## Author Contributions

MH: participated in research design, performance of the research, data analysis and in the writing of the paper. JW, JM, PF, CB, MK, BG, HB, and FM: participated in performance of the research, interpretation of data for the work and in the revising of the paper. MM: participated in performance of the research, interpretation of data for the work and in the writing of the paper. LR: participated in research design, in the performance of the research, interpretation of data for the work, and in the writing of the paper. All authors contributed to the article and approved the submitted version.

## Funding

This work was supported in part by the Intramural Research Program of the NIH, the National Institute of Environmental Health Sciences (ZIAES101074), the Myositis Association, and the Centers for Disease Control and Prevention (grant 1H75DP001743-01). Social and Scientific Systems was supported under a contract with NIEHS (HHSN273201600011C). Efforts from HB and MM were supported by the National Institutes of Arthritis and Musculoskeletal Skin Diseases under Award - Number P30AR076316.

## Conflict of Interest

JW and JM were employed by DLH Holdings Corp Company. PF was a contractor with Kelly Government Solutions. The remaining authors declare that the research was conducted in the absence of any commercial or financial relationships that could be construed as a potential conflict of interest.

## Publisher's Note

All claims expressed in this article are solely those of the authors and do not necessarily represent those of their affiliated organizations, or those of the publisher, the editors and the reviewers. Any product that may be evaluated in this article, or claim that may be made by its manufacturer, is not guaranteed or endorsed by the publisher.
