# Supplementary material for: The Geospatial Distribution of Myositis and Its Phenotypes in the United States and Associations With Roadways: Findings From a National Myositis Patient Registry
Source: Front Med (Lausanne). 2022 Mar 16;9:842586. doi: 10.3389/fmed.2022.842586 (PMC8966380; doi:10.3389/fmed.2022.842586)
Supplement: Supplementary file 1 [file Data_Sheet_1.docx]

**Supplementary Methods**

**Major and minor road networks**

The major road network contains the primary roads that are generally divided, limited-access highways within the interstate highway system, and is distinguished by the presence of interchanges. The minor road network contains the secondary roads that are main arteries, usually in the U.S. Highway, State Highway, or County Highway system.

**Statistical analysis**

*Finding clustering patterns in myositis incidences:* Inhomogeneous *J*-function (1, 2) is commonly used in spatial point process to characterize the distribution of points after adjusting for an area characteristics such as population density. When the distribution of points is completely at random, the spatial point process is generated from a Poisson point process and the *J*-function will be a flat line at one for any distance, $r$, between pair of points. The point patterns are regular (or, inhibition) when $J_{inhom}\left(r\right)>1$, and clustering when $J_{inhom}\left(r\right)<1$. It is essential to determine the correct patterns in the spatial distribution of myositis cases, which can guide selecting appropriate spatial point process models. The inhomogeneous *J*-function in Figure 3 is consistently less than 1, indicating that the myositis cases in the U.S. and its subgroups have clustering patterns.

*Orientation of myositis cases:* To examine whether in any direction there is higher concentration of cases existed in the myositis prevalence for the entire U.S. as well as by subgroups, we used the nearest-neighbor orientation density methods (3). This method considers each point of a myositis case, finds its nearest neighbor, and measures an angle in degrees anti-clockwise from the x-axis for the joining line. A kernel estimate of the probability density of the angles is calculated and denoted as .

*Relative risk by subgroups:* In examining whether there exist any spatial trends in the increased prevalence of cases of subgroups such as DM vs PM, or IIM cases with lung disease vs cases without lung disease, we used a recently proposed symmetric adaptive smoothing scheme for estimating spatial relative risk (4). The method employed an improved version of spatially adaptive bandwidths selection methods to handle the extensive inhomogeneity in the distribution of the data such as the myositis cases.

*Space-time separability Test:* To investigate the separability hypothesis of the intensity function of the spatio-temporal point process, i.e., whether the intensity function of the spatio-temporal point process can be expressed as the product of the intensity functions of spatial point process and temporal process, we used nonparametric tests as proposed by Schoenberg (5) and the independence by nearest-neighbor indices (6). Among the nearest-neighbor indices, we used normalized nearest-neighbor mean and variogram, and nearest-neighbor correlation from a mark point process, where the quantitative marks are the year of diagnosis. Values close to one for normalized nearest-neighbor mean and variogram, and close to zero for nearest-neighbor correlation indicate independence of marks.

Tests of separability between the spatial point and temporal processes using the Schoenberg (5) tests for the entire U.S. myositis cases were not conclusive. Because of this inconclusive evidence, we examined the independence of year of diagnosis marks using the nearest-neighbor mean and variogram, and nearest-neighbor correlation. These measures were 0.994, 0.997, and 0.021, respectively, indicating the marks to be independent in the distribution of points. Thus, we considered a spatial point process for the entire study period.

*Tests for the interdependence between qualitative marks:* The myositis cases have various subgroups, including adult DM, adult PM, IBM, as well as patients with and without lung disease based on patient report by questionnaire. It is of interest to know whether these subgroups can be treated independently when measuring the effect of distance in the subgroup analyses. We made all possible pairwise comparisons for the distribution of points for the patients in the subgroups DM, PM and IBM, and for the pair with and without lung disease by using the mark connection function (6). Pairwise mark connection function $p_{ij}\left(r\right)$ is the conditional probability, measures the correlation between two marks $i$ and $j$, which are apart by distance $r$. Comparing the behavior of the mark connection function $p_{ij}\left(r\right)$ with the case when the marks $i$ and $j$ are independent (i.e., $2p_{i}p_{j}$), will indicate whether the marks at distance $r$ were assigned independently (i.e., random labelling). The estimated mark connection functions for the pairs: DM and PM, DM and IBM, PM and IBM, and with and without lung disease are given in supplement Figure S4. Each mark connection function (solid line) for the increasing distance between subgroups is contained within the bounds created by Monte Carlo simulation (gray shading), indicating the spatial distribution of subgroups are independent. This justified examining the spatial characteristics as well as developing separate models for each subgroup while assessing the exposure effect, in addition to all IIM.

*Spatial point process models:* Spatial point process methods provide a flexible modeling framework for spatially explicit information, such as the spatial locations of myositis cases, and spatially defined covariates, such as the population characteristics in the same locations where the cases occur, as well as the population characteristics where no case occurred by using dummy points. Although the estimates can be sensitive to the number of dummy points, for a relatively large number of points, the estimates are generally stable. These methods also allow incorporating discrete or continuous variables characterizing the events, known as marks, in a joint modeling framework. For example, the myosotis subtypes can be considered as discrete marks and the age of myositis diagnosis as continuous marks. In our applications of spatial point process models, we assessed the effects of covariates independent of marks.

In autoimmune diseases, clustering of cases is common. After examining the spatial distribution of cases with myositis and its subgroups, the *J*-functions indicated the presence of clustering patterns. Among the models that deal with clustering, the log-Gaussian Cox process (LGCP) (7) is popular due to its simplicity and mathematical tractability. We used LGCP models with population density as an offset value for assessing the effect of distance to roads on intensity of myositis cases. The covariates that are included in the LGCP model are of three types: distance to road as a categorical variable with three levels, the location of points as latitude and longitude values, and all remaining spatially varying variates as pixel images. We examined the sensitivity of results for different choices of covariance structures such as exponential or Matern. The results were consistent, and we preferred to report the results from LGCP model with Matern covariance structure.

References

1. Van Lieshout M, Baddeley A. A nonparametric measure of spatial interaction in point patterns. Statistica Neerlandica. 1996;50:344-61.

2. Van Lieshout M. A J-function for inhomogeneous point processes. Statistica Neerlandica. 2011;65:183-201.

3. Illian J, Penttinen A, Stoyan H, Stoyan D. Statistical Ananlysis and Modelling of Spatial Point Patterns: Wiley; 2008 2008.

4. Davies T, Jones K, Hazelton M. Symmetric adaptive smoothing regimens for estimation of the spatial relative risk function. Computational Statistics & Data Ananlysis. 2016;101:12-28.

5. Schoenberg F. Testing separability in spatial-temporal marked point processes. Biometrics. 2004;60:471-81.

6. Stoyan D, Stoyan H. Fractals, random shapes and point fields: methods of geometrical statistics: John Wiley and Sons; 1994.

7. Benes V, Bodlak K, Moller J, Waagepetersen R. A case study on point process modelling in disease mapping. Image Analysis and Stereology. 2005;24:159-68.

**Supplementary Figures.**

**Figure Legend**

**Figure S1**: Counts of IIM cases by diagnosis year.

**Figure S2**: The estimated mark connection function in solid line of various pairs of marks (i.e., subgroups). A) Dermatomyositis and Polymyositis; B) Dermatomyositis and Inclusion body myositis; C) Inclusion body myositis and Polymyositis; and D) IIM cases without and with lung disease. In all plots, dashed lines represent subgroups that are independent, and the gray shadings are the pointwise envelopes for the independence of subgroups from 99 Monte Carlo simulations.

**Figure S3:** Empirical J-function (solid line) and log-Gaussian Cox process model fitted J-function (dashed line) with 95% confidence band in gray shading for: A) all IIM; B) Dermatomyositis; C) Polymyositis; D) Inclusion body myositis; E) IIM cases with lung disease, and F) IIM cases without lung disease.

**Figure S1:**


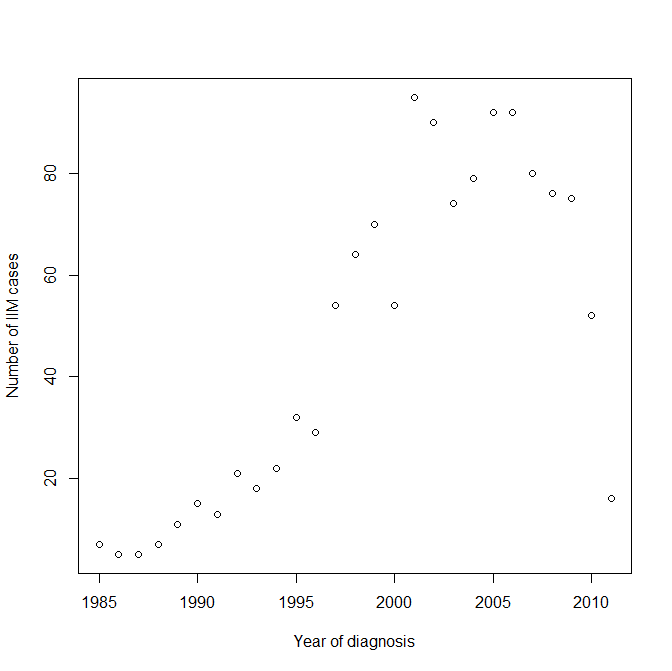


**Figure S2:**


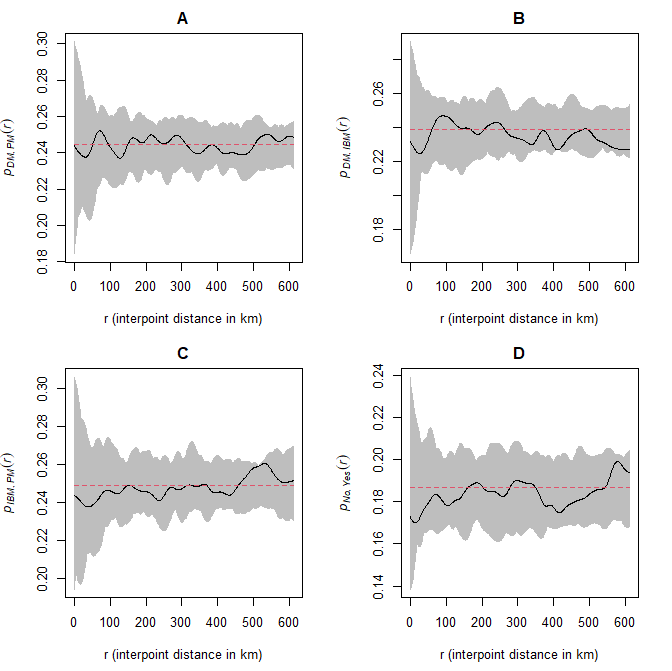


**Figure S3:**


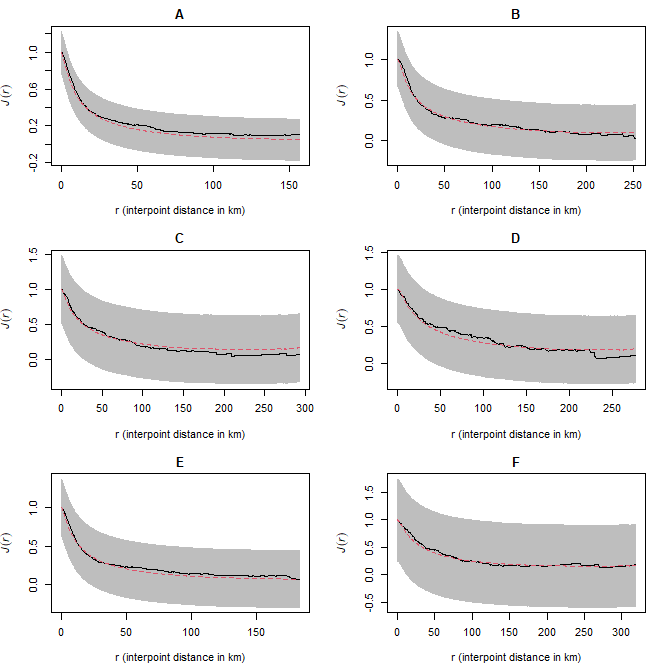


**Supplementary Tables.**

**Table S1:** Peaks in anticlockwise direction from horizontal (east) axis (in degree) of U.S. myositis cases and its subgroups using nearest neighbor orientation density method

|  | Peak (in degree) |
| --- | --- |
| All IIM | 63.4 |
| DM | 24.0 |
| JDM and DM | 19.0 |
| PM | 292.4 |
| JPM and PM | 291.7 |
| IBM | 357.2 |
| With lung disease | 8.5 |
| Without lung disease | 336.8 |

**Table S2:** Coefficient estimates (with 95% CI) of covariates used for adjustment in each log-Gaussian Cox process regression model.

| Covariate | All IIM | DM | PM | IBM | Lung disease | No lung disease |
| --- | --- | --- | --- | --- | --- | --- |
| Longitude | 0.00005 (-0.0002, 0.0004) | 0.00003 (-0.0003, 0.0004) | 0.00008 (-0.0003, 0.0004) | 0.00004 (-0.0002, 0.0003) | 0.00002 (-0.0003, 0.0003) | 0.00005 (-0.0003, 0.0004) |
| Latitude | -0.00003 (-0.001, 0.001) | -0.0002 (-0.001, 0.001) | 0.0002 (-0.001, 0.001) | -0.0001 (-0.001, 0.0008) | -0.0002 (-0.001, 0.0008) | -0.000004 (-0.001, 0.001) |
| White percent^#^ | 0.07 (-3.26, 3.40) | 0.27 (-3.57, 4.10) | 0.02 (-3.83, 3.87) | 0.18 (-2.96, 3.31) | 0.44 (-2.96, 3.83) | 0.12 (-3.49, 3.73) |
| Female percent^#^ | 43.21 (4.60, 81.82)* | 45.37 (0.62, 90.13)* | 45.29 (-0.96, 91.54) | 35.56 (-1.16, 72.29) | 49.99 (10.49, 89.49)* | 43.01 (1.00, 85.03)* |
| Age 7-17 percent^#^ | -79.03 (-177.61, 19.56) | -88.24 (-200.33, 23.86) | -95.25 (-212.70, 22.20) | -36.76 (-128.94, 55.41) | -58.37 (-163.03, 46.28) | -83.93 (-187.90, 20.05) |
| Age 18-44 percent^#^ | -42.24 (-103.28, 18.80) | -44.00 (-114.56, 26.55) | -51.35 (-124.10, 21.41) | -22.24 (-81.4, 36.93) | -21.14 (-86.16, 43.88) | -47.16 (-111.81, 17.48) |
| Age 45-64 percent^#^ | -38.12 (-88.41, 12.17) | -39.75 (-97.80, 18.31) | -45.17 (-104.07, 13.74) | -21.12 (-69.94, 27.70) | -20.88 (-74.96, 33.21) | -42.74 (-95.61, 10.13) |
| Age ≥ 65 percent^#^ | -47.82 (-109.42, 13.77) | -50.99 (-121.69, 19.71) | -59.22 (-133.48, 15.04) | -23.74 (-82.75, 35.27) | -32.27 (-97.91, 33.38) | -51.37 (-116.75, 14.01) |
| MH Income | -0.06 (-0.57, 0.45) | -0.03 (-0.61, 0.55) | -0.05 (-0.67, 0.57) | -0.15 (-0.62, 0.32) | -0.007 (-0.52, 0.50) | -0.07 (-0.62, 0.48) |
| High school graduate percent^#^ | 1.94 (-7.12, 11.01) | 1.06 (-9.13, 11.25) | 2.02 (-9.12, 13.16) | 3.18 (-5.08, 11.45) | 2.75 (-6.34, 11.83) | 1.65 (-8.09, 11.40) |
| College graduate percent^#^ | 2.40 (-6.42, 11.22) | 2.56 (-7.75, 12.87) | 0.39 (-10.33, 11.11) | 3.29 (-5.01, 11.59) | -1.01 (-1.01, 8.11) | 3.51 (-6.04, 13.05) |
| RUCA | -0.22 (-0.41, -0.03)* | -0.22 (-0.44, 0.003) | -0.23 (-0.47, 0.002) | -0.23 (-0.42, -0.05)* | -0.23 (-0.43, 0.03)* | -0.21 (-0.41, 0.003) |
| Smoking percent^ | 0.04 (-0.09, 0.17) | 0.05 (-0.10, 0.19) | 0.03 (-0.13, 0.19) | 0.03 (-0.09, 0.15) | 0.04 (-0.09, 0.18) | 0.05 (-0.09, 0.19) |

*p-value≤0.05; **p-value≤0.01; ^#^Percentage in 0 to 1 scale; ^Percentage in 0 to 100 scale

Abbreviations: IIM, idiopathic inflammatory myopathies; DM, adult dermatomyositis; PM, adult polymyositis; IBM, inclusion body myositis; m, meters; CI, confidence interval
